# More than eight in every nineteen inmates were living with depression at prisons of Northwest Amhara Regional State, Ethiopia, a cross sectional study design

**DOI:** 10.1186/s12888-016-1179-9

**Published:** 2017-01-19

**Authors:** Teresa Kisi Beyen, Abel Fikadu Dadi, Berihun Assefa Dachew, Niguse Yigzaw Muluneh, Telake Azale Bisetegn

**Affiliations:** 1 Department of Public Health, College of Health Sciences, Arsi University, Asella, Ethiopia; 2 Department of Epidemiology and Biostatistics, Institute of Public Health, College of Medicine and Heath Science, University of Gondar, Gondar, Ethiopia; 3 Department of Psychiatry, School of Medicine, College of Medicine and Heath Science, University of Gondar, Gondar, Ethiopia; 4 Department of Health Education and Behavioral Science, Institute of Public Health, College of Medicine and Heath Science, University of Gondar, Gondar, Ethiopia

**Keywords:** Depression, Inmates’ depression, Internees’ depression, Depression in custody

## Abstract

**Background:**

Mental health is the greatest challenges for the current and future generations. Worldwide, out of the 66 million people suffering from depression; majority (85%) were from low and middle income countries. The prevalence was more common among the prisons population than the community. However, a worldwide consideration given to the problems is very low, particularly for prisoners.

**Methods:**

To assess level of depression and associated factors among prisoners in prisons of Northwest Amhara Regional State, Ethiopia, Institutional based cross sectional study was employed on 727 prisoners selected by multistage random sampling from three prisons of northwest Amhara. Patient Health Questionnaire (PHQ-9) was used to assess an individuals’ depression level. The reliability of the tool was checked by Cronbach’s Alpha (yielding value of 0.841). Multivariable logistic regression was done to identify factors associated with depression after Hosmer and lemeshow goodness of fit test was used for model fitness.

**Results:**

Of the total prisoners participated (649), 284 (43.8%; 95% CI: 39.90, 47.67%) had symptoms of depression. Detainees’ satisfaction level about life before imprisonment, belief about their life after imprisonment, plan to commit suicide, social support and types of prisons were significantly associated with depression.

**Conclusions:**

Depression level among detainees was found to be high. Thus, providing training to scale up satisfaction of prisoners, on how to cope up with environment just before imprisonment and release, and treating prisoners will improve the problem.

## Background

Worldwide, there are about 10 million people in prisons. Worldwide, prison population is being raised by around one million per decade. The majority of the world prison population were observed at low- and middle-income countries [1]. According to Penal Reform International (PRI) 2015 report, since 2004, the size of the world prison population has increased by approximately 10%. Accordingly, Over the last 15 years, prison populations have indicated sharp rises by 150, 125 and 53% in Brazil, Colombia and Mexico respectively. US showed 16% increment in between 2001 and 2012, while also in Asia steep rises have been seen particularly in Indonesia (183%), Vietnam (136%), and China (modest rise). In Europe, since 2000, prison populations have fallen in Russia (particularly in Baltic States) and in some Eastern countries (e.g. Romania) even though it began to rise again after 2010. In UK and France increments have also been seen continuously. In Africa, while data are less completed, large percentages of increment have been seen in some Northern African countries like Algeria (i.e. it was 76% between 2001 and 2013) and Morocco. In South Africa prison numbers was raised to maximum in 2004 and then decreased to 158,000 in 2014. The number of detainees have been risen in some, but not in all, East African countries; from 55,000 in 2000 to 93,000 in 2011 in Ethiopia [2].

Estimated 450 million people worldwide suffered from mental or behavioral disorders [3]. The problems were especially prevalent in prison populations [4]. About 11% of prisoners world-wide were suffering from common mental health problems such as depression and anxiety [5]. Mental health presents one of the greatest health problem that current and future generations will face [6]. Epidemiological studies conducted among prisoners in many countries have shown a high prevalence of psychiatric morbidity. The magnitude of severe mental disorders was five to ten times higher among prisoners compared to the general population [7]. Other studies added that mental problem was more common among the prison population [4, 8]. In European prisons, the prevalence of psychotic disorders was about 5%, depressive or anxiety disorders was estimated to be 25%, and substance-related disorders was approximately 40% [9] while study among women’s prison in Sa˜o Paulo revealed that the prevalence of common mental disorders was reported as 26.6% [10]. Many of these disorders might be present before admission to prison, and might be further exacerbated by being detained [11, 12]. However, mental disorders might also be developed during imprisonment itself as a result of prevailing conditions, possibly due to torture or other human rights violations [12].

World Health Organization (WHO) forecasted, in 2001, that by 2020 depression will be the second leading contributor to the global burden of disease [13]. Additionally, according to the 2009 discussion paper released by World Health Organization, out of 66 million people suffering from depression; 85% live in low and middle income countries [14]. An extensive literature review done in 24 countries revealed rates of depression of around 10 and 14% in males and females prisoners respectively [15]. Many studies reported different levels of depression among detainees; 46.1% among Norwegian inmates [16], 59.4% among incarcerated women in Central Prison of Peshawar, Pakistan [17], 29% among sentenced prisoners in Iran [18], 18% among prisoners of England and Wales (including anxiety) [19], 10 and 12% among men and women prisoners respectively [20], 23.3% among prisoners of Durban, South Africa (including psychotic and anxiety disorders) [21], and 49% among prisons and jails according to special reports of U.S. department of Justice [22]. In Kaliti Federal Prisons, Addis Ababa, Ethiopia, 61.9% of prisoners were found to have high levels of mental distress in general [23].

There were many factors in prisons that have contribution on mental health, particularly on depression including; overcrowding, various forms of violence, enforced solitude or conversely, lack of privacy, lack of meaningful activity, isolation from social networks, insecurity about future prospects (work, relationships, etc.), inadequate health services, especially mental health services in prisons, luck of social support, dissatisfaction before and after imprisonment, older ages and status of prison. The increased risk of suicide in prisons (often related to depression) was, unfortunately, one common manifestation of the cumulative effects of these factors [11, 12, 24–28].

In Ethiopia, approximately 1.7% of the national health expenditure was spent on mental health in 2004. So, in order to tackle mental health problem the Government of Ethiopia launched a National based Mental Health Strategy that can enable the government to deliver comprehensive and integrated service to mental health needs of Ethiopians [29, 30].

Majority of the population in the prison was found in the productive age category that will be returned to their community after they complete the time at jail. The government gives high attention to the prisoners to create productive mentality through implementation of different strategies that lead to the production of entrepreneur prisoners of the future country. However, the emphasis given to mental health was very low across the globe in general and for prisoners in particular. This is even more in countries with limited resource and still there is no accurate magnitude of prisoners with mental disorder who were incarcerated in Ethiopia, particularly in Northwest of Amhara Regional state and information about prisoners’ health conditions is scarce. Even though health care service for mental disorder was designed in the national health policy of Ethiopia, interventions against the problem were very limited, which might be due to limited information about the problem. Thus, establishing the prevalence rates of mental disorders, particularly depression, is of great importance [31]. As a result, this study aimed to assess magnitude of depression and its attributes among prisoners detained in prisons of Northwest Amhara regional state, Ethiopia which will serve as an input for policy makers, health service planners and strategy designers.

## Methods

### Study design and area

Institution based cross sectional study was conducted to determine the magnitude of depression and associated factors among prison inmates found in the prisons of North West Amhara regional state, Ethiopia, from January to February 2015. The region is one of the 11 regions found in the Democratic Republic of Ethiopia. The region covers a total area of 20,650,420 Km^2^ with a total population of 19,602,512. There were 30 prisons in the region of which 10 found in the North West part of Amhara region. The numbers of prisoners found in 30 prisons were 22,590 while 7564 prison inmates were detained in the selected prisons.

### Sample size and sampling procedure

Multi stage random sampling technique was used to select 727 detainees for the study. Three prisons (i.e. Bahirdar, Debre tabor and Gondar prisons) were randomly selected by lottery method from the ten prisons found in the Northwest Amhara Regional state. Then, the sample size was proportionally allocated to each prison. Then after, computer generated random number was used to select the required samples from each prisons using openEpi software. Thus, all prisoners found in the selected prisons of the Northwest Amhara regional state were the study populations. Those prisoners who were seriously ill and unable to communicate were excluded from the study. The optimum sample size (n) was computed by single population proportion formula [n = [(Z_a/2_)^2^*P (1-P)]/d^2^] by assuming 95% confidence level, 5% margin of error (d), design effect of two, 61.9% proportion (p) among Kality prisoners [23].

### Data collection and data quality control

Data were collected using structured interview aided questionnaire having seven parts (i.e. Socio-demographic characteristics, Generalized Anxiety Disorder 7-item (GAD-7) (scale ranging from zero (not at all) to three (nearly every day)) [32], Kessler Psychological Distress Scale (K10) with five level response, Patient health questioner (PHQ-9), used to assess an individual’s depression scale [33, 34], social support (measured by Orientation of Social Support (OSS), scaled from 1 (very strongly disagree) to 7 (very strongly agree)) [35], Suicidal ideation and attempt, and behavioral factors, which includes history of substance use). Patient health questionnaire (PHQ-9) which contained nine questions each measuring a problem that the prisoners bothered in the last 15 days were used to measure depression with scale measurement ranging from zero (not at all) to three (nearly every day). Receiver operating characteristic (ROC) curve analysis was done by STATA version12 software in order to determine a cut off value with high sensitivity and specificity. An individual was considered as in the state of depression if he/she has a score above seven (cut off value) which provided ROC curve area of one with *p*-value of < 0.001. The sensitivity and specificity of the tool was found to be the highest at cut off value of seven. The internal consistency of the tools was checked by Cronbach’s Alpha which yielded 0.841 values for over all internal consistency; with inter-item correlation ranging from 0.31 to 0.59 and Cronbach’s alpha if items deleted ranging from 0.85 to 0.86. The questionnaire was pre-tested before actual data collection and collected by eight B.Sc. holders after training was delivered for them on how to collect data. Then, the collected data were reviewed and checked for completeness before data entry and incomplete data were considered as none response rate.

### Data processing and analysis

Data were coded, cleaned (through checking incomplete questionnaires during data collection, by doing frequency distribution and graphical presentation) and entered to Epi Info 7 and imported to STATA version 12 for further analysis. Both descriptive and inferential biostatistics procedures were undergone. Tables were used to present the data. Both bivariable and multivariable logistic regression model were used to identify factors associated with depression. Adjusted odds ratio with its 95% Confidence interval and *p*-value was used to determine the final model. The variables were entered to the multivariable model using forward likelihood ratio variable selection method. Model fitness was tested by Hosmer and lemshow goodness of fit which provided *p*-value of 0.75 and minus log likelihood, which reduced from 889.569 to 786.198 providing chi-square of 103.371 with *p*-value of less than 0.001.

## Results

### Prisoners’ socio demographic characteristics

Out of total sample size, 649 (90%) of them responded completely to the interview. The median age of the study participants was 27.75 years with inter-quartile range (IQR) of 11.7 years. Majority of the internees were males (89.8%), 66.9% were from urban, most of them (90%) were orthodox followers, about half (47.1%) of them were unmarried and 32% were grade nine to 12 complete (Table 1).

**Table 1:** Socio demographic characteristic of prisoners in the prisons of northwest Amhara Regional State, Ethiopia, January-February, 2015 (*n* = 649)

Covariate	Frequency (%)
Sex	
Male	583 (89.8)
Female	66 (10.2)
Residence	
Urban	434 (66.9)
Rural	215 (33.1)
Religion	
Orthodox	584 (90)
Others^a^	65 (10)
Marital status	
Single	306 (47.1)
Married	228 (35.1)
Not live with partners	115 (17.7)
Educational status	
Not read and write	108 (16.6)
Read and write	97 (14.9)
1–8 grade complete	129 (19.9)
9–12 grade complete	206 (31.9)
Certificate and above	109 (16.8)

^a^Muslim, Catholic and Protestant

### Characteristic of prisoners

The median year of stay in the penitentiary, of study participants, was 9.3 years with IQR of 3.7 years. About 22% of the inmates were sentenced for life. About half of the inmates spent most of their time on religious practices and 60% of the study participants engaged in income generating activities in the prisons. Only 10% of the study participants responded that they were satisfied with the care provided in the penitentiary (Table 2).

**Table 2:** Characteristics of prisoners in the prisons of Northwest Amhara Regional State, Ethiopia, January to February, 2015 (*n* = 649)

Covariates	Frequency (%)
Type of prisoners	
Life time prisoners	138 (21.3)
Not life time prisoners	511 (78.7)
Religious practice	
Always	308 (47.5)
Sometimes	229 (35.3)
Never	112 (17.3)
Participate in income generating activities	
Yes	389 (59.9)
No	260 (40.1)
Having job before being prisoner	
Yes	467 (72)
No	182 (28)
Felt happy before being prisoner	
Yes	567 (87.4)
No	82 (12.6)
had friend in the prison	
Yes	407 (62.7)
No	242 (37.3)
Discriminated because of imprisonment^a^	
Yes	283 (43.6)
No	365 (56.4)
Frequency of feeling guilty of crime	
Always	354 (54.5)
Sometimes	105 (16.2)
Never	190 (29.3)
Perceived magnitude of mistake	
Hard	304 (46.8)
Medium	152 (23.4)
Low	193 (29.7)
Accepted crime committed	
Yes	267 (41.1)
No	313 (48.2)
No idea	69 (10.6)
Crime penalty accepted	
Yes	30 (4.6)
No	557 (85.8)
No idea	62 (9.6)
Satisfaction with the care in the prison	
Good Satisfaction	65 (10)
Medium satisfaction	580 (89.4)
Low satisfaction	4 (0.6)

^a^by friends, parents, and relatives

### Social support, and suicidal ideation and attempt

Out of total internees, 293 (45.1%) were without social support of which 9.9% were females. Nearly 17% of the total internees reported that they had idea of committing suicide since their imprisonment and 16.6% have already planned to commit suicide. Additionally, 11.9% of them reported that they have made at least one attempt of suicide since imprisoned. The most reported method for the attempt of suicide were hanging (45.5%) followed by using poison (31.2%) while majority of them reported that they attempted suicide since they became hopeless due to the crime they have committed (39.0%), due to economic problem (15.6%) and felt guilty of the crime committed (18.2%) (Table 3).

**Table 3:** Social support, and suicidal ideation and attempt of prisoners in the prisons of Northwest Amhara Regional State, Ethiopia, January to February, 2015, (*n* = 649)

Variables	*n* (%)
Had idea of committing suicide	
Yes	110 (16.9)
No	539 (83.1)
Planed to commit suicide	
Yes	108 (16.6)
No	541 (83.4)
Suicide attempted	
Yes	77 (11.9)
No	572 (88.1)
Methods attempted (*n* = 77)	
Hanging	35 (45.5)
Poison	24 (31.2)
Use sharp tools	18 (23.4)
Reasons for attempting suicide (*n* = 77)	
Family conflict	6 (7.8)
Economic problem	12 (15.6)
Death of family	4 (5.1)
Feel guilty of crime	14 (18.2)
Hopelessness due to crime	30 (39.0)
Lack of social support	11 (14.3)

### Prisoners’ mental health and substance abuse

Out of the total study participants, 284 (43.8%; 95% CI: 39.90, 47.67%) showed signs of depression. About 14% of the prisoners reported that they had previous history of psychiatric problem and only 12.9% showed up that one of their families had experienced mental illness. About 33% of the study participants had a feeling of impossibilities to run the life they had before when released from the custody. Nearly 17 out of 20 (83.4%) prisoners were victims of psychological distress while seven of every 20 (36.1%) prisoners were at risk of anxiety. About 13 out of 20 detainees were wishing excuse of their crime and 35.3% of the detainees reported that they had no social support. Nearly, 5% of prisoners were current smokers while 18.2% of prisoners had history of Khat chewing, Using Shisha, Cigarette smoking and/or Alcohol drinking.

### Factors associated with depression

Bivariable logistic regression found that marital status, type of sentenced prisoners, satisfaction with day to day activity before imprisonment, discrimination due to crime, acceptance of crime penalized for, previous psychiatric problem, having family members with mental illness, thinking impossibility not to run the life they had before, social support, thinking to commit suicide, having plan to commit suicide, type of prisons and attempting suicide were significantly associated with depression. However, by Multivariable logistic regression only satisfaction with day to day activity before imprisonment, thinking impossibility not to run the life they had before, social support, type of prison and plan to commit suicide found to be significantly associated with depression (Table 4).

**Table 4:** Factors associated with depression by bivariable and multivariable logistic regression among prisoners in the prisons of Northwest Amhara regional state, Ethiopia, January to February, 2015 (*n* = 649)

Explanatory variables	Depression		COR, 95% CI	AOR, 95% CI	*P*-value
	Yes	No			
Marital Status^a^					
Married	88	140	0.82 (0.58, 1.16)		
Divorced	24	28	1.12 (0.62, 2.01)		
Separated	20	15	1.74 (0.86, 3.52)		
Widowed	19	9	2.75 (1.20, 6.26)		
Single	133	173	1		
Type of sentenced prisoner ^a^					
Life time	72	66	1		
Not life time	212	299	0.65 (0.45, 0.95)		
Satisfaction of day to day life before imprisonment					
Yes	232	335	0.40 (0.25, 0.65)	**0.44 (0.26, 0.73)**	**0.002**
No	52	30	1		
Discriminated^a^					
Yes	145	138	1.72 (1.25, 2.35)		
No	139	227	1		
Accepted crime^a^					
No	126	187	1		
Yes	113	154	1.09 (0.78, 1.52)		
Don’t know	45	24	2.78 (1.61, 4.80)		
Previous psychiatric problem^a^					
Yes	58	34	2.50 (1.58, 3.94)		
No	226	331	1		
Family history of mental illness^a^					
Yes	46	38	1.66 (1.05, 2.64)		
No	238	327	1		
Impossibilities to run life as before					
Yes	124	89	**2.40 (1.72, 3.33)**	**1.87 (1.30, 2.69)**	**0.001**
No	160	276	1		
Thought committing suicide^a^					
Yes	81	29	4.62 (2.92, 7.31)		
No	203	336	1		
Had plan to commit suicide					
Yes	81	27	4.99 (3.13, 7.98)	**4.16 (2.56, 7.77)**	**0.000**
No	203	338	1		
Attempted suicide since imprisoned^a^					
Yes	56	21	4.02 (2.37, 6.83)		
No	228	344	1		
Social support					
Yes	133	223	0.56 (0.4, 0.77)	**0.62 (0.44, 0.87)**	**0.006**
No	151	142	1		
Place of the prison					
Gondar	94	125	0.62 (0.43, 0.89)	**1.54 (1.04, 2.29)**	**0.034**
Debre Tabor	60	130	0.38 (0.26, 0.57)	**2.27 (1.46, 3.51)**	**0.000**
Bahir Dar	130	110	1		

^a^significant only by bivariable logistic regression

Variables that have *p*-value less than 0.05 in multivariable logistic regression were considered as significant

## Discussion

This study disclosed the level of depression and associated factors among prisoners in the prisons of Northwest Amhara regional state. The study revealed that more than eight out of 19 internees were identified with depression (43.8%). The result goes with the reports of study done among Norwegian inmates (46.1%) [16]. This result also in line with results reported by studies conducted on different types of populations, a systematic literature review of depression among Australian women, which reported prevalence ranging from 2.6 to 43.9% [36], report’s of study done in Hamadan, Iran among population over 65 years old (48.3%) [37], and in Netherland; among older persons (48.4%) [38]. However, It is higher than results of study in Iran among sentenced prisoners (29%) [18], reports of Bureau of Justice Statistics among State prisoners (23%) and jail inmates (30%) [22], systematic review of 62 studies from 12 countries; which reported 10% among men and 12% among females [20], study conducted in Agaro town (15%) [39], in low- to middle-income countries which ranges from 5.9 to 11.1% [28], and northern Uganda (29.2%) [40]. On the other hand it is lower than results reported on Woman in Central Prison, Peshawar, Pakistan (59.4%) [17], by studies done in Germany among general adults [41]. The possible explanation for the differences might be socio-demographic, socio-economic and cultural difference between our study population and the listed studies. There were also measurement (like cut off value, and tool difference) and prison status difference which might be the other possible explanations.

The study showed that detainees who were not satisfied with their day to day life before imprisonment were 56% more likely to show signs of depression when compared to their counterpart [AOR = 0.44; 95% CI: 0.26, 0.63]. In line with this study, many studies among different population suggested that satisfaction had strong association with depression; which stated strong positive association between low satisfaction and depression [42]. Aligned with this, respondents who thought that they would face difficulty of running life as before after being free of imprisonment were 47% more likely to develop depression when compared to their counterpart [AOR = 1.87; 95% CI: 1.30, 2.69]. The possible reason could be as the prisoners worry about their future life they become more depressed; they are also the most stigmatized segment of the population in the society because of the crime they have done previously.

On the other way, the odds of developing depression among prisoners who had plan to commit suicide were more than four times more likely when compared with prisoners who hadn’t plan to commit suicide [AOR = 4.16; 95% CI: 2.56, 6.77]. This finding in lined with earlier reports of world health organization and American Psychiatric Association which showed that mental health disorders (specially depression) were related with more than 90% of all cases of suicide [43] and major depressive disorder alleviate the risk of suicidal ideation, attempted suicide and death by completed suicide [25]. A study conducted on inmates of New South Wales, Australia also confirmed this association [44]. Another study also showed the evidence of strong positive association between depression and suicide [40]. However, prisoners who had social support were 62% less likely to be with depression’s signs when compared to those who hadn’t social support [AOR = 0.62; 95% CI: 0.44, 0.89]. Many studies on the different population showed that depression was high among individuals who had poor social support. The possible reasons stated were lack of (poor) social support which may lead to increased psychological distress; on the other hand, good social support is vital for the prevention of anxiety, both of which have relation with depression [45]. Other studies added that loneliness has adverse consequences for mental health including depression [40, 46–49].

The study showed that prisoners in the Gondar and Debre Tabor prisons were more likely to be imitated by depression when compared to Bahir Dar prison with [AOR = 1.54; 95% CI: 1.04, 2.29] and [AOR = 2.27; 95% CI: 1.46, 3.51] respectively. This finding was strengthened by the result from Jos maximum Security Prison, Plateau State which indicated a strong association between depression and status of prison [27]. The possible explanation for this could be age distributions of the prisoners as the distribution of old ages were higher in the Gondar and Dabre Tabor prisons. Even though age is not associated to depression in our study; studies supported that depression were more likely to occur among old ages [28, 36, 40, 49–55]. The other possible reason could be as Bahir Dar’s prison is regional level; there may be facility difference, which might improve the satisfaction level of the prisoners.

Even though the study indicated very important factors associated with depression, the study is not free of the limitations of cross sectional study design like lack of indicating the strong cause and effect relationship. Additionally, the study is not still free of social desirability bias because subjects were systematically more likely to provide a socially acceptable response since data was collected through self report. Furthermore, the study did not collect information on the injury and trauma as they might be other factors associated with depression.

## Conclusion

In conclusion, depression level among detainees in the region was significantly high. Prisoners who had satisfaction with their day to day life before being imprisoned and social support were less likely to have depression while those who had a plan to commit suicide and who thought that they will have impossibility to run life as before if released from the prison were more likely to have depression. Additionally, place of prisons also associated with depression.

### Recommendation

It would have been better if the government as well as the administrators of each prison strengthen social support within each prison and support of relatives, peers, and families for the prisoners. In addition, providing training to scale up satisfaction of prisoners, on how to cop up with new environment just before imprisonment and release, on suicide reduction and treating prisons with psychological distress and depression improve depression level.

## Abbreviations

AOR: Adjusted odds ratio
COR: Crude odds ratio
GAD-7: Generalized anxiety disorder 7-item
IQR: Inter-quartile range
K10: Kessler psychological distress scale
OSS: Orientation of social support
PHQ-9: Patient health questionnaire
PRI: Penal reform international
ROC: Receiver operating characteristic
WHO: World Health Organization
